# Bio-based phenolic branched-chain fatty acid in wash water reduced populations of *Listeria innocua* on apple fruit^☆^

**DOI:** 10.1016/j.heliyon.2024.e24901

**Published:** 2024-01-17

**Authors:** Victor Ryu, Piyanan Chuesiang, Joseph Uknalis, Helen Ngo, Tony Jin, Xuetong Fan

**Affiliations:** a USDA, ARS, Eastern Regional Research Center, 600 East Mermaid Lane, Wyndmoor, PA, 19038, USA; b Department of Food Technology, Faculty of Science, Chulalongkorn University, Bangkok, 10330, Thailand

**Keywords:** Phenolic branched-chain fatty acids, Antimicrobial, Apple wash, Sanitizer, *Listeria innocua*, *E coli*, Post-harvest

## Abstract

Phenolic branched-chain fatty acid (PBC-FA) emulsion was produced by dissolving it in ethanol and mixing with water (pH 7). The resulting monodispersed emulsion droplets were approximately 200 nm in diameter. The stability of the emulsion was evaluated by storing it at 4 and 20 °C for 30 days. The antimicrobial activity of the PBC-FA emulsion was tested against *Escherichia coli* and *Listeria innocua* (8 log CFU/mL) by determining the minimum inhibitory concentration (MIC) and minimum bactericidal concentration (MBC) using a microdilution method. The PBC-FA was effective against *L. innocua* with MIC and MBC of 14.1 μg/mL and caused membrane permeation as determined with SEM and Live/Dead cell assay, but was not effective against *E. coli* O157:H7 at the tested concentrations (5–250 μg/mL). We also evaluated PBC-FA emulsion's potential to be used as a wash against *L. innocua* inoculated on apples. The results showed that the 500 μg/mL PBC-FA emulsion with 5 % ethanol had equivalent antimicrobial activity (2–3 logs reductions) against *L. innocua* as the 20 μg/mL chlorine solution, a commonly used sanitizer. 500 μg/mL PBC-FA emulsion had better antimicrobial efficacy when organic matter (chemical oxygen demand: 9.0 g/L) was present compared to 20 μg/mL of chlorine. The effect of PBC-FA on the quality of the apples, was determined by measuring changes in color, firmness, and soluble solids content over a 14-day storage period at 20 °C. The quality of the apples was not affected by PBC-FA over the 14-day storage period, suggesting that PBC-FA emulsion can be used as a wash for apples without affecting their quality.

## 1 Introduction

Washing produce is an important step in reducing microbial loads and removing debris and soil from the surface of fruits and vegetables[1]. One study reported that more than half of the *Listeria* spp*.* found in different sites of the produce packaging facility was *L. monocytogenes*, and it was mostly found in drain, cold storage, and wet food contact surface sites [2]. Listeriosis outbreaks and recalls on caramel apples and apple slices due to contamination of *L. monocytogenes* have been reported in 2015 and 2017 [3,4]. The surface of the apple has many microstructures that could potentially harbor or protect the bacteria from sanitizers, such as waxy cuticles, microcracks, lenticels, and trichomes [4]. In a previous study, researchers found that washing apples with chlorine water resulted in varying degrees of bacterial protection on the surface of the fruit [5]. The structures that provided the most to the least protection were the floral tube, lenticels, damaged cuticle surrounding the puncture wounds, and intact cuticle [5].

Chlorine is the most common sanitizer used by the food industry to sanitize water and minimize cross-contamination [6]. When washing produce, chlorine should be applied less than 2000 μg/mL and should be rinsed to remove the residues according to the 21 Code of federal regulations part 173 (CFR21. Part 173) [7]. Up to 200 μg/mL chlorine is commonly used in wash water for produce with a contact time of 1 min or greater often in multiple wash steps [6,7]. Their antimicrobial efficacy depends on the pH, as they work best at pH 6.5–7.5 but can become corrosive at pH levels below 6 and generate chlorine gas below pH 5 [7]. Two main byproducts formed during chlorination are halogenated trihalomethanes and haloacetic acid, which are toxic and are associated with an increaed risk of getting cancerdue to exposures at high levels [8]. Its antimicrobial efficacy also decreases when organic compounds are present [1,9]. Studies have shown that bacteria treated with chlorine could become resistant to disinfectants and antibiotics [10,11]. Some studies have observed that there could be a higher transfer of plasmid-encoded antibiotic-resistant genes due to reactive oxygen species (ROS) oxidative stress on bacteria induced by chlorine or chloramine [12,13]. Therefore, the emergence of chlorine-resistant bacteria due to inadequate sanitation could also become resistant to antibiotics.

One of the alternative antimicrobial compounds that could be used is fatty acid-based derivatives, as fatty acids are relatively stable, active at low pH, don't create harmful byproducts, and are non-corrosive [14]. Also, many of these compounds have low acute toxicity and are approved to be used as food additives or classified as “generally recognized as safe” (GRAS) [[15], [16], [17]]. Lastly, some fatty acids could reduce the possibility of the emergence of drug-resistant bacteria as they inhibit bacteria by interacting with multiple targets sites, causing permeation of membrane, disruption of membrane, enzyme activity, electron transport chain, and uncoupling of oxidative phosphorylation [14,18]. It was also reported that bacteria are not likely to mutate or develop resistance when exposed to antimicrobial lipids [18]. Recently, Ngo et al. [19], reported the production of phenolic branched-chain fatty acid (PBC-FA) using phenol, oleic acid, and a modified H^+^ ferrierite zeolite catalyst [19]. However, when this compound (PBC-FA) was tested against bacteria, they found that it was only effective against Gram-positive bacteria and not against Gram-negative bacteria [20].

In this study, size, net surface potential, and stability of PBC-FA spontaneous emulsion in different storage temperatures were measured. The spontaneous emulsion is produced by mixing two immiscible liquids that are not in equilibrium without external energy [21]. Emulsification of lipophilic antimicrobial compounds improve their dispersibility and increase the surface area, thereby allowing more compounds to interact with the bacteria in an aqueous system. Also, the mechanism of PBC-FA's antimicrobial activity against *Listeria innocua* (*L. innocua*), a common surrogate of *L. monocytogenes* and *Escherichia coli* (*E. coli*) O157:H7 was investigated using SEM and Live/Dead cell assay. We also determined whether PBC-FA could be used as an alternative antimicrobial agent to chlorine in apple wash water. The antimicrobial efficacy of PBC-FA emulsion and chlorine against *L. innocua* on the apple was determined before and after removing the residue. The effect of organic matter on the antimicrobial efficacy of PBC-FA emulsion and chlorine was tested as well. Also, the effect of PBC-FA emulsion and chlorine on the quality of the apples was compared during the 14 days of storage. This study provides insight into whether PBC-FA emulsion could be effective as chlorine when used as wash water for produce.

## 2 Materials and methods

### 2.1 Materials

Phosphate buffered saline (PBS) was made by mixing sodium chloride (S9888-500g, Sigma-Aldrich, St. Louis, MO, USA), sodium phosphate dibasic (S907-500g, Sigma-Aldrich), and sodium phosphate monobasic (7892, Sigma-Aldrich). 200-proof ethanol was purchased from Koptec (64-17-5, King of Prussia, PA, USA). 20 μg/mL chlorine was prepared from 7.5 % sodium hypochlorite (Chlorox Com., Aberdeen, MD, USA) and confirmed using DR/890 colorimeter (HACH, Loveland, CO, USA) and DPD free chlorine reagent (Permachem Reagent, Loveland, CO, USA). PBC-FA was synthesized using the same method as described by Fan et al. [20]. Unwaxed Gala apples were purchased from an orchard near Morgantown, PA USA.

### 2.2 Stock PBC-FA emulsion

A 1000 μM PBC-FA stock emulsion was made by first dissolving the PBC-FA in ethanol, then diluted in PBS with pH of 7, resulting in the final concentration of ethanol being 5 %. The PBC-FA stock was then stored at 4 or 20 °C.

### 2.3 Stability of stock PBC-FA emulsion

To determine the mean particle diameter (Z-average) and polydispersity index (PDI) of the emulsion, dynamic light scattering was employed. The Zetasizer blue pro (model ZSU3200, Malvern Instrument, UK) was utilized to capture the back-scattered laser intensity fluctuations, and the instrument's software (Zsxplorer, Malvern Panalytical) was employed to calculate the distribution of particle size. Furthermore, particle electrophoretic light scattering was conducted to assess the net surface charge (ζ-potential). Throughout a storage period of 30 days at 4 or 20 °C, the size and surface charge of the PBC-FA stock emulsions were monitored every 7 days.

### 2.4 Bacterial culture conditions

*E. coli* O157:H7 (ATCC-700728) and *L. innocua* (ATCC-33090) were obtained from the American Type Culture Collections (Manassas, VA). They were stored in cryopreservative solution (MicrobankTM 2D, Pro-Lab Diagnostics, Roundrock, TX, USA) at −80 °C in cryovials to preserve the cultures. Working stocks were created by streaking the cultures onto selective media: MacConkey Sorbitol Agar (279,100, BD Diagnostic Systems, Berkshire, UK) for *E. coli* and PALCAM Agar (222,530, BD Diagnostic Systems) with the antimicrobial supplement for *L. innocua*, and kept at 4 °C and used for two weeks. Overnight cultures were prepared by inoculating a colony from the working stock into tryptic soy broth (TSB; BD Diagnostic Systems) and incubating it at 37 °C on a 100 rpm shaker for 24 h. For the antimicrobial assay, the optical density at 600 nm (OD600) of a 1:10 dilution of the 24 h cultures of *E. coli* O157:H7 and *L. innocua* was adjusted to 0.17 cm^−1^ and 0.14 cm^−1^, respectively, to confirm that the culture contained approximately 9 log CFU/mL. The bacteria were washed once with 0.1 % peptone (218,071-500g, BD Diagnostic Systems) and centrifuged at 4000 g for 10 min. The OD600 was measured again to confirm that there was no significant loss of bacteria during the washing step. To achieve an initial inoculum of approximately 8 log CFU/mL for treatment, the number of bacteria was confirmed using serial dilution and spread plating on Tryptic Soy Agar (TSA; Neogen, Lansing, MI, USA).

### 2.5 Antimicrobial assay

The minimal inhibitory concentrations (MICs) and minimal bactericidal concentrations (MBCs) were determined using a microdilution method with slight modification according to the method from Fan et al. [20]. 100 μL of different concentrations of PBC-FA emulsion was diluted in 96 well microtiter plate containing 100 μL of TSB. Then 20 μL from the overnight culture which has approximately 9 log CFU/mL was added to the well so that the initial inoculum was 8 log CFU/mL. The final concentration of ethanol during the antimicrobial assay was approximately 2.5 %. The 96 well microtiter plate with the compounds was incubated for 24 h at 37 °C, with shaking at 100 rpm. After that, 20 μL of 0.1 % (w/v) thiazolyl blue tetrazolium bromide (M2128-1g, Sigma-Adrich) was added and MICs were determined as the lowest concentration of test compound that did not lead to visible color changes (no active growth). MBCs were determined by spread plating the contents of the wells that showed no growth followed by incubation of the plates at 37 °C for 24 h. The lowest concentration yielding no bacterial colonies was recorded as the MBC.

To study the growth pattern of *L. innocua* in the presence of PBC-FA to confirm the MIC from the above assay, OD600 of the samples was measured at 1 h intervals using a microplate reader (Synergy H1, Biotek Instrument Inc., Santa Clara, CA, USA) at 37 °C with 30 s of shaking throughout a 24 h period. The data was collected and exported using imager software (Gen 5, version 3.11, Biotek Instrument Inc).

#### 2.5.1 Scanning electron microscopy (SEM)

For sample preparation, 50 μL of bacteria were placed onto acetone-cleaned 12 mm Micro-cover glass slides, allowing them to adhere for 30 min. The bacteria were then fixed with 2 mL of 2.5 % glutaraldehyde for an additional 30 min. The fixed samples were subsequently rinsed twice for 30 min each with 2–3 mL of 0.1 M imidazole and then with 50, 80, and 90 % ethanol solutions (2–3 mL each) for 30 min each. The samples were then washed three times with 2 mL of 100 % ethanol and critically point dried. The dried samples were stacked in a wire basket, separated by cloth, and subjected to further drying using liquid carbon dioxide for approximately 20 min in a critical point drying apparatus. Finally, the samples were mounted on stubs, sputter gold-coated for 1 min, and imaged using a FEI Quanta 200 F Scanning Electron Microscope (Hillsboro, OR, US) with an accelerating voltage of 10 KV in high vacuum mode.

### 2.6 Live/dead cell assay

To quantify the percentage of cells with a permeable membrane, a live/dead cell assay was carried out. The Live/Dead™ BacLight™ bacterial viability kit (L7012) was used to mix 1.5 μL of Syto 9 and propidium iodide in a centrifuge tube. A total of 3 μL of the dye mixture was added to 1 mL of a 24 h treated bacterial suspension with a concentration of 8 log CFU/mL which was washed with 0.1 % peptone water. The resulting mixture was incubated in the dark at room temperature for 15 min, after which 10 μL of the sample was placed on a 35 mm poly-d-lysine coated dish (P35GC-1.4-14-C). A confocal microscope (Leica DMI 4000B, Wetzlar, Germany) was used to capture fluorescence signals of the cell-permeable dyes, with excitation of the dyes performed using a 488 nm laser. The micrographs obtained were subsequently analyzed using Image J 1.53k software (National Institutes of Health, Bethesda, MD, USA) to determine the proportion of cells exhibiting a permeable membrane, which was indicated by the emission wavelength detected from the propidium iodide dye.

### 2.7 Antimicrobial assay on apple

Colony from a working stock was inoculated in 500 mL TSB and incubated at 37 °C for 24 h on a shaker at 100 rpm to obtain a solution with 9 log CFU/mL. An aliquot of 300 mL of the inoculum was centrifuged at 4000 g for 10 min at 4 °C. The pellets were resuspended in 0.1 % peptone water. The refrigerated apples were held at room temperature for an 1 h (2 apples per treatment) prior to the wash in 200 μg/mL chlorine for 1 min and rinsed twice with distilled water. The apples were, then, dried for 2 h in the biosafety hood on the sterilized rack. The inoculum was diluted by adding 300 mL inoculum to 2700 mL of PBS pH 7 solution, and then apples were dip inoculated by submerging them in the agitated bacteria solution for 5 min. The inoculated apples were dried again for another 2 h in the biosafety hood on the sterilized rack. To apply treatments to apples, an apple was submerged in a beaker containing 500 mL of treatment solution. A weight was placed on top of the apples to keep them from floating and the solution was agitated on a shaker at 50 rpm for 1 min. The cells were resuspended by manual massaging and agitating the stomacher bag (BagFilterP 111,200, Interscience, France) containing 250 mL 0.1 % peptone water on the vortex at the maximum level for 3 min. After this, the apple was taken out from the stomacher bag and the second apple was submerged inside the stomacher bag and cells were resuspended using the same method. The resuspended culture on the other side of the membrane in the stomacher bag was serially diluted and spread plated on TSA and PALCAM, before incubating the plates at 37 °C for 48 h.

Also, to determine whether the residue of antimicrobials left on the apple affected the assay, we conducted a separate antimicrobial assay by introducing a rinsing step after treating the apple with PBC-FA or chlorine. The treated apple was submerged in PBS for 30s before placing it into 250 mL of peptone water in a stomacher bag.

### 2.8 Cell injury

The percentage of injured *L. innocua* on the apple after treatments were calculated using Eq (1) by comparing the number of colonies on selective (PALCAM) and nonselective (TSA) agar [[22], [23], [24]].

(1) $$\left[1-\frac{\#\;of\;colonies\;on\;selective\;agar}{\#\;of\;colonies\;on\;nonselective\;agar}\right]*100$$

### 2.9 Effect of organic load

The organic load for the experiment consisted of autoclaved apple juice, which was extracted using a MAR-48C juicer (Plastaket MFG Co. Inc., Lodi, CA, USA). The chemical oxygen demand (COD) of apple juice in solution was determined using high-range COD digestion vials (2415915, HACH, Loveland, CO, USA) and DR/890 colorimeter. A 0.5 mL of apple juice was mixed with 9 mL of either chlorine or PBC-FA. Subsequently, *L. innocua* was inoculated into the mixture to achieve a final bacteria concentration of 7 log CFU/mL. *L. innocua* was treated for 5 min and was spread plated on TSA.

### 2.10 Quality of apple

Apples for quality analysis were not inoculated but treated similarly as for antimicrobial assay. After treatments, color, hardness, and BRIX values of treated apples were measured every 7 days during 14 days of storage at 20 °C with relative humidity of 70 %. There were three replicates of apples with 2 fruits for each replicate on each sampling day.

#### 2.10.1 Color

The surface color of each piece of apple was determined using an UltraScan VIS spectrophotometer (Hunter Associates Lab, Reston, VA, USA), which was calibrated using standard black and white plates. On each sampling day, two apples were used for each replicate of the treatment, and two measurements were taken from the shade and blush parts of each apple [25]. The Commission Internationale de l'Eclairage (CIE) color, L*, a*, b*, and ΔE values were recorded using the instrument's software (EasyMatch QC ver. 4.89, Hunter Associates Lab).

#### 2.10.2 Hardness

To evaluate the firmness of the apple texture, a Texture Analyzer XT Plus (Texture Technologies Corp, South Hamilton, MA, USA) was used. A probe with a diameter of 11 mm was placed at the center of each peeled site of the apple, and it penetrated 10 mm at a speed of 10 mm/s. Two apples were used for each replicate of the treatment, and two measurements were taken on each sampling day. The peak positive force and positive area were recorded for each measurement using the instrument's software (Exponent software ver. 6.1.4.0, Texture Technologies Corp).

#### 2.10.3 Brix

The apple juice was extracted using a MAR-48C juicer. The Brix value, a measure of dissolved solid substance, was determined using a Refractometer AR200 (13950000, Reichert, Germany).

### 2.11 Data acquisition and analysis

All experiments were carried out in triplicate. The statistical analyses were performed using *p* ≤ .05 to represent statistical significance using Statistical Package for the Social Sciences software (IBM SPSS Statistics, Version 16, IBM, USA). The differences in the average diameter of emulsion and polydispersity index (PDI) between day 1 and day 30 of storage were determined by paired *t*-test. An independent *t*-test was used to assess the differences in the average droplet size and PDI between PBC-FA emulsion stored at 4 and 20 °C. The differences between average color, peak positive force, positive area, Brix values, and log reductions after treatments were evaluated using Duncan's new multiple range test.

## 3 Results and discussion

### 3.1 Stability of PBC-FA emulsion

PBC-FA emulsion was a spontaneous emulsion as it was produced in isothermal conditions, and it didn't require any external energy to produce the emulsion. The average diameter of the emulsion was around 206 ± 3.8 nm, and it had PDI of 0.08 ± 0.02, which indicates that it was monodispersed (Table 1). Storage stability of PBC-FA emulsion was assessed by measuring changes in the average size and PDI of the emulsion droplet during the storage period of 30 days at 4 and 20 °C. The average diameter of the emulsion droplet increased over the 30 day storage regardless of the temperature they were stored in, which indicated that they were not thermodynamically stable. However, the emulsion droplets were still monodispersed (Table 1, Fig. S1-Supplementary Material) and there was no indication of creaming or phase separation.

**Table 1:** Mean diameters, polydispersity indexes (PDI), and zetapotential of PBC-FA emulsion on day 1 and day 30 after storage at 4 or 20 °C. The numbers are means ± standard deviation (n = 3).

Treatment	4 °C		20 °C	
	Day 1	Day 30	Day 1	Day 30
Z-average (nm)	206 ± 3.8^a^	226 ± 10^Ab^	206 ± 3.8^a^	227 ± 2^Bb^
PDI	0.08 ± 0.02^a^	0.09 ± 0.02^Aa^	0.08 ± 0.02^a^	0.1 ± 0^Ba^
Zeta potential (mV)	−55 ± 0.8^a^	−59 ± 6^Aa^	−55 ± 0.8^a^	−56 ± 2^Aa^

a Different capital letters (p ≤ .05) were used to indicate significant differences between treatments on corresponding days, while different lower-case letters were used to indicate significant differences (p ≤ .05) between means on days within the treatment.

The net surface charge of the droplet was indicated by ζ-potential. The ζ-potential of the emulsion droplet was around −55 ± 0.8 mV, and it didn't change during the 30 day storage period at both temperatures (Table 1). The net negative charge may have been due to the carboxyl group (carboxyl group has pKa around 5) of PBC-FA as most of the emulsion surface will be constituted of hydrophilic carboxyl groups of PBC-FA for oil-in-water emulsion [18]. The pKa of phenol in water was reported to be 9.99 in water [26]. However, in the previous paper, phenol was predicted to be in the carbon chain of PBC-FA, and it may have had a lesser influence on the surface charge of the emulsion droplet [20]. Therefore, since the pH of PBC-FA emulsion was at 7, the deprotonation of the carboxyl group on the surface of the emulsion droplet may have been the reason for having a net negative charge.

### 3.2 Antimicrobial assay of PBC-FA emulsion

The MIC and MBC against *L. innocua* and *E. coli* O157:H7 were determined using colorimetric assays with thiazolyl blue tetrazolium bromide and by measuring OD600 of the bacteria for 24 h at 37 °C. 2.5 % ethanol in the solution did not affect the growth of either *L. innocua or E. coli* O157:H7. Freshly produced and 30 days old PBC-FA emulsions stored at 4 and 20 °C were used, and their MIC and MBC were compared. The PBC-FA emulsion's antimicrobial efficacy remained the same since there were no changes in MIC and MBC against both bacteria (Table 2) or growth profiles (Fig. S2). Similar to the previous result, PBC-FA had no effect against *E. coli* O157:H7, even at a higher concentration than before[20]. To determine the mechanism of how PBC-FA inhibits the growth of *L. innocua*, SEM and Live/Dead cell assay was used.

**Table 2:** MIC and MBC of PBC-FA emulsion against 8 LogCFU/mL of *L. innocua* and *E. coli* O157:H7 on day 1 and day 30 after storage at 4 or 20 °C.

Bacteria	Treatment	4 °C				20 °C			
		Day 1		Day 30		Day 1		Day 30	
		MIC (μg/mL)	MBC (μg/mL)	MIC (μg/mL)	MBC (μg/mL)	MIC (μg/mL)	MBC (μg/mL)	MIC (μg/mL)	MBC (μg/mL)
*L. innocua*	Ethanol	>25,000	>25,000	>25,000	>25,000	>25,000	>25,000	>25,000	>25,000
	PBC-FA	14.2	14.2	14.2	14.2	14.2	14.2	14.2	14.2
*E. coli* O157:H7	Ethanol	>25,000	>25,000	>25,000	>25,000	>25,000	>25,000	>25,000	>25,000
	PBC-FA	>455	–	>455	–	>455	–	>455	–

### 3.3 SEM micrographs

**Fig. 1:**
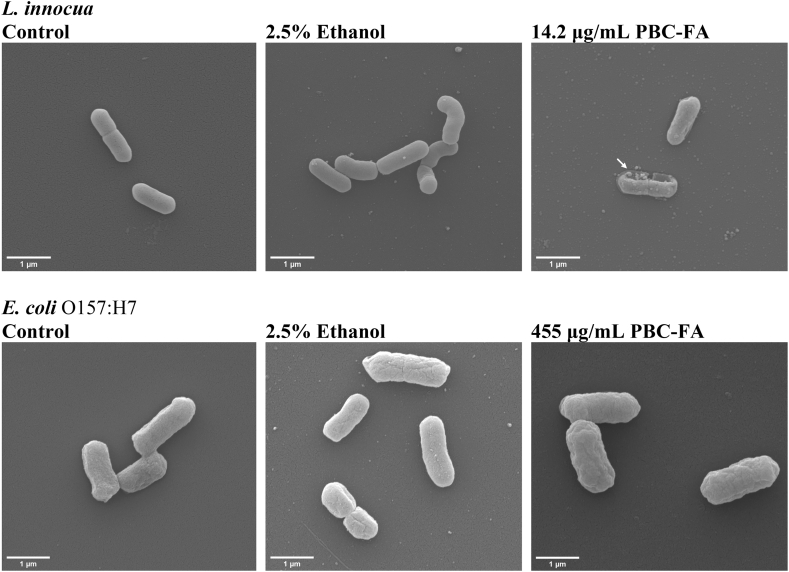
Representative SEM micrographs of treated *L. innocua* and *E. coli* O157:H7 with PBC-FA emulsion. The white arrow indicates damage to the cellular membrane. The white arrow indicates the disruption of the membrane.

SEM micrographs of *L. innocua* and *E. coli* O157:H7 treated with PBC-FA emulsion at MIC are shown in Fig. 1. The micrographs indicated that there was an irregular cellular surface on the *L. innocua* after it was treated with PBC-FA emulsion (Fig. 1). For *E. coli* O157:H7, the cells looked intact after it was treated with PBC-FA emulsion.

### 3.4 Live/dead cell assay

**Fig. 2:**
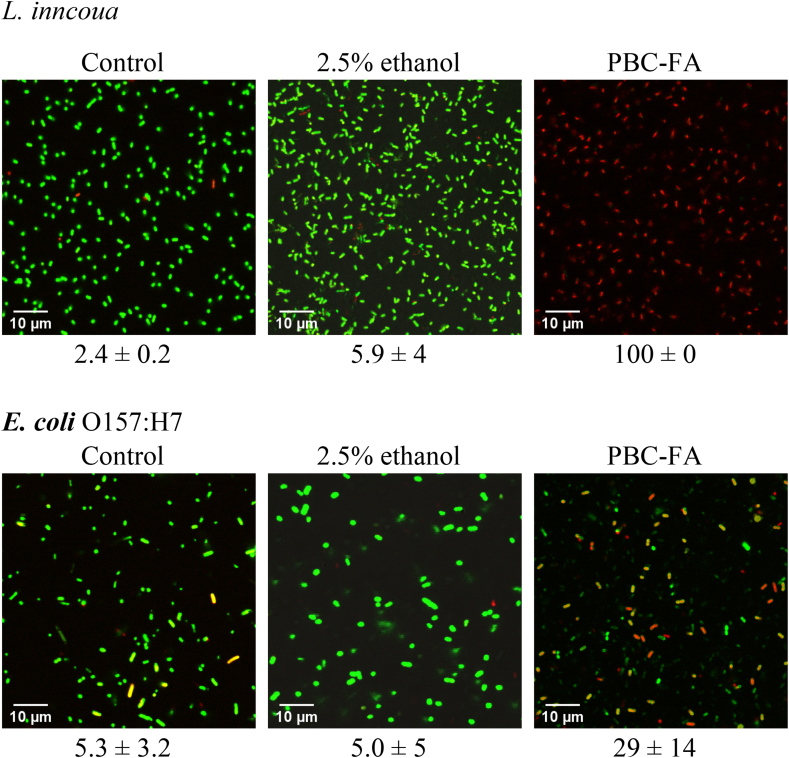
Representative micrographs of *L. innocua* and *E. coli* O157:H7 treated with PBC-FA emulsion stained with Syto9 and propidium iodide. The percentage of *L. innocua* and *E. coli* with permeable membranes (red) is listed below each micrograph. (For interpretation of the references to color in this figure legend, the reader is referred to the Web version of this article.)

Live/Dead cell assay was conducted on *L. innocua* and *E. coli* O157:H7 treated with PBC-FA at MIC. Similar to the results from the SEM micrographs, *L. innocua* had a high number of cells with permeable membrane, while *E. coli* O157:H7 had a relatively lower percentage of cells with permeable membrane (Fig. 2). Gram-negative bacteria are known to be more resistant to antimicrobial agents than Gram-positive bacteria due to their cell envelope structure which includes an outer membrane containing lipopolysaccharides (LPS) and porins. The LPS layer acts as a physical barrier that restricts the access of hydrophobic compounds, such as toxins or antibiotics, to the inner membrane and cytoplasm [27]. Furthermore, the porins mediate passive diffusions and could regulate the integrity of the membrane [28]. Previous study has reported that Gram-negative bacteria exhibit higher resistance to antimicrobial fatty acids than Gram-positive bacteria [29]. However, the exact mechanisms underlying this difference in susceptibility remain to be fully elucidated.

Gram-positive and Gram-negative bacteria have a negative charge on the surface due to teichoic acid and LPS in the membrane. It has been reported that the charge density on the membrane of Gram-negative bacteria could be different as compared to Gram-positive bacteria. For instance, compared to *Lactobacillus rhamnosus*, *E. coli* had seven times larger negative charge density on the surface [30]. Therefore, the intensity of electrostatic repulsion between the PBC-FA emulsion droplet and *E. coli* O157:H7 may have been stronger compared to *L. innocua*.

### 3.5 Antimicrobial activity of PBC-FA emulsion against *L. innocua* on apple

**Fig. 3:**
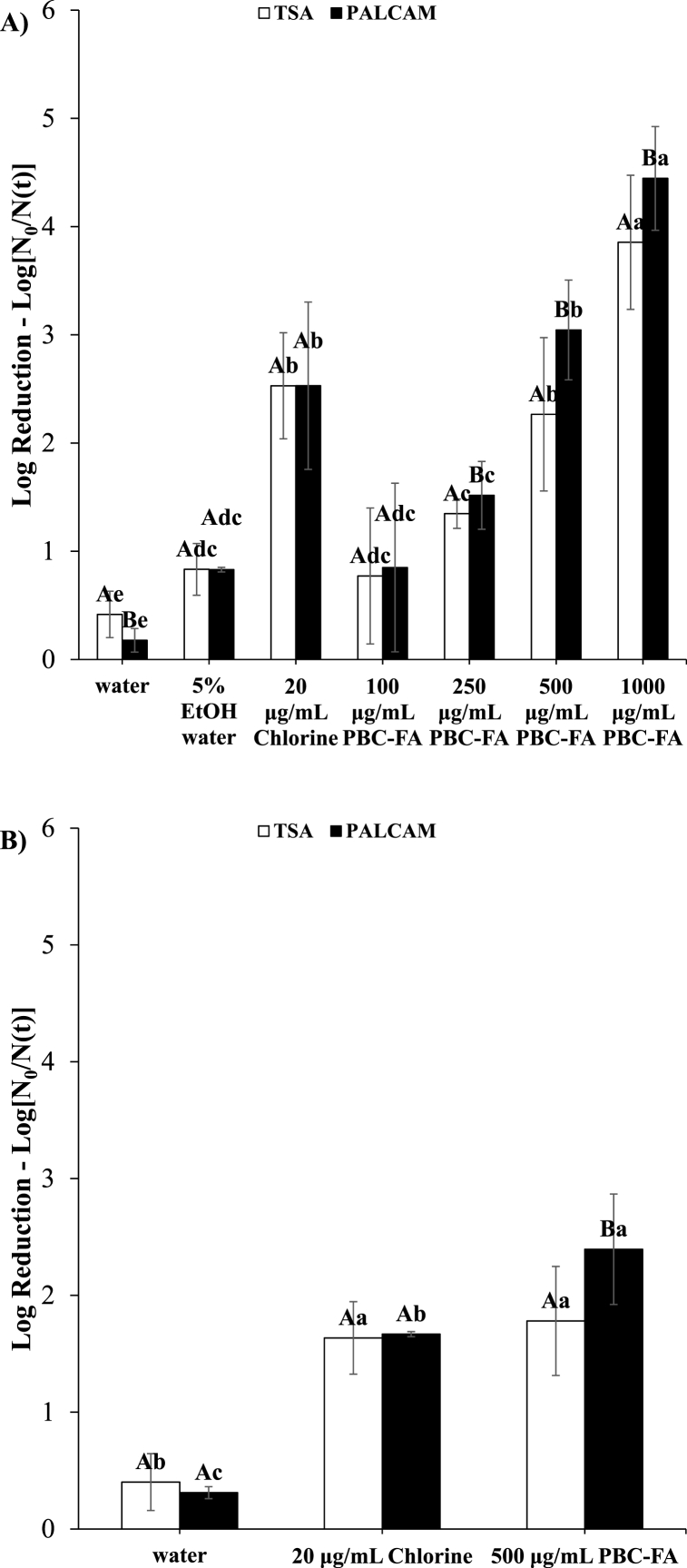
Reductions in counts of *L. innocua* treated with chlorine or PBC-FA emulsion A) before and B) after the rinsing step. Capital letters indicate differences between colony reductions on TSA and PALCAM. Lower-case letters indicate differences in the reduction of colonies among treatments on TSA or PALCAM. Means with the same letters are not significantly different (p > .05). Vertical lines represent standard deviation of means (n = 3).

To determine the concentration of PBC-FA emulsion that has equivalent antimicrobial efficacy against *L. innocua* contaminated apples compared to 20 μg/mL chlorine used as wash water, different concentrations of PBC-FA emulsion were tested. Washing apples with water alone or 5 % ethanol water reduced the population of *L. innocua* by less than 1 log CFU/mL. 500 μg/mL PBC-FA had a similar antimicrobial capacity as 20 μg/mL chlorine against *L. innocua* on contaminated apples, achieving about 3 log reductions (Fig. 3). However, the percentage of injured cells differed when *L. innocua* was treated with 20 μg/mL chlorine (5.4 ± 3.4) compared to when it was treated with 500 μg/mL PBC-FA (32.6 ± 22.8) (Fig. 3). The higher level of injury caused by PBC-FA emulsion may be due to its hydrophobicity, which allows it to be adsorbed onto the apple peel which is covered with hydrophobic wax and wax platelets [4]. After soaking the apple in different treatment solutions, the weight of the apple increased the most for the one dipped in PBC-FA emulsion (Table S1).

To determine whether the antimicrobial activity of 20 μg/mL chlorine and 500 μg/mL PBC-FA emulsion was not due to the residue that remained after the treatment, the treated apple was rinsed for 30 s in PBS. The antimicrobial efficiency decreased after the rinsing step for both treatments (Fig. 3) indicating residues do have effect on the antimicrobial assay. However, even after adding the rinsing step, it was shown that the antimicrobial capacity of 20 μg/mL chlorine and 500 μg/mL PBC-FA emulsion were similar (Fig. 3). The % injury of cell after the treatment of *L. innocua* was statistically similar between 20 μg/mL chlorine (5.1 ± 9.1) and 500 μg/mL PBC-FA (25.4 ± 18.3) samples (Fig. 3). Therefore, 500 μg/mL PBC-FA emulsion had a similar antimicrobial capacity as 20 μg/mL chlorine solution.

We tested neutralizing buffer to replace peptone water for the inactivation of residual sanitizers after the wash step. The neutralizing buffer neutralized 20 μg/mL chlorine but not PBC-FA (data not shown), indicating that the mechanism of the inactivation of bacteria by PBC-FA was not due to oxidation. The 20 μg/mL chlorine and 500 μg/mL PBC-FA emulsion absorbed in the apple was around 0.4–0.5 g (Table S1). It was determined that diluting the treated apple in 250 mL of 0.1 % peptone water in the stomacher bag sufficiently reduced PBC-FA concentration to levels below the MIC. Therefore, the reductions of *Listeria* by PBC-FA were not due to the residual effect of the sanitizer.

### 3.6 Effect of organic load on the antimicrobial activity of PBC-FA emulsion against L. innocua

**Fig. 4:**
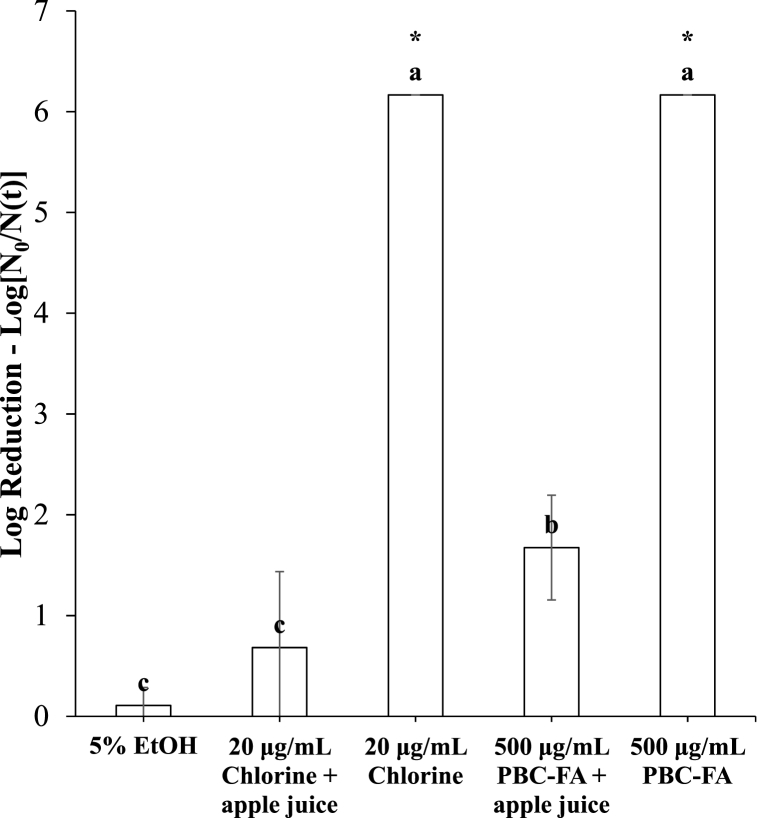
Reductions in counts of *L. innocua* treated with chlorine or PBC-FA emulsion in the presence of organic matter (apple juice). Lower-case letters indicate differences in the number of reductions in colonies between treatments. Means with the same letters are not significantly different (p > .05). “*” indicates that the log reduction was below the limit of detection. Vertical lines represent standard deviation of means (n = 3).

To assess the impact of organic load on the antimicrobial efficacy of 20 μg/mL chlorine and 500 μg/mL PBC-FA emulsion against 7 log CFU/mL of *L. innocua*, 0.5 mL of apple juice was added prior to bacterial inoculation. The COD value of solutions with apple juice was 9007 ± 382 mg/L. In the absence of apple juice, both 20 μg/mL chlorine and 500 μg/mL PBC-FA emulsion achieved a reduction of 6 log CFU/mL *L. innocua* (populations below the limit of detection 1.47 log/mL) within 5 min. However, the presence of apple juice significantly decreased their antimicrobial efficacy (Fig. 4). When apple juice was added, the antimicrobial efficacy of 500 μg/mL PBC-FA emulsion was better than that of 20 μg/mL chlorine (Fig. 4). This may be due to the different mechanism of action that PBC-FA and chlorine take to inactivate the bacteria as mentioned in the previous section.

### 3.7 Quality of apple after washing with PBC-FA emulsion

In order to assess the impact of PBC-FA emulsion on apple quality, the apples were deliberately not rinsed after treatment. PBC-FA contains a phenolic group that could potentially scavenge oxygen, and we previously investigated whether it could act as an antioxidant agent. However, our findings indicated that the antioxidant activity was not significant (data not shown). Despite this, we evaluated the quality of the treated apples by measuring changes in their color, hardness, and soluble solid content.

Gala apple has a color of red, yellow, and shaded colors, which is due to different concentrations of anthocyanin, carotenoid, and chlorophyll [31]. It is an indicator of the maturity of the apple and whether it is harvestable or not [31]. There was no clear trend of changes in L* and a* values of apples during the storage period. b* value had no indication of whether PBC-FA emulsion caused an increase in its value during storage (Table S2). Nevertheless, the b* value at the end of storage for both shade and blush sides of the apple was the highest among all treatments, indicating the yellowing of apples during simulated shelf-life. Hardness (peak value and area under curve) and BRIX value had no significant differences during the storage period among all treatments (Tables S3 and 4). Therefore, 20 μg/mL chlorine and 500 μg/mL PBC-FA emulsion had no effect on the quality of the apple during the 14-day storage period at 20 °C.

Overall, PBC-FA emulsion has shown a potential for use as a sanitizer in wash water for apples to reduce microbial populations without compromising fruit quality. However, commercial use of PBC-FA emulsion as a chlorine replacement in wash water is limited as it could not reduce Gram-negative bacteria. Nevertheless, the novel sanitizer provides a tool for the produce industry to minimize pathogen contamination during postharvest handling of apples considering *Listeria* being a common pathogen associated with fresh and fresh-cut apples [3].

## 4 Conclusions

The production of PBC-FA emulsion with droplets of approximately 200 nm in diameter was achieved by dissolving it in ethanol and pouring it into PBS at pH 7. The stability of the emulsion was evaluated and found to be maintained after storage at both 4 °C and 20 °C for 30 days. The PBC-FA emulsion exhibited antimicrobial activity against *L. innocua* through membrane permeation but not against *E. coli*. Interestingly, the PBC-FA emulsion at a concentration of 500 μg/mL was found to be equally effective as a wash water against *L. innocua* on apples, compared to the 20 μg/mL chlorine wash solution. The efficacy of 500 μg/mL PBC-FA emulsion was less affaced by the presence of organic matter than chlorine. Moreover, PBC-FA did not affect the quality of the apples, as evidenced by the lack of significant changes in color, hardness, and soluble solids content over a 14-day storage period at 20 °C. Overall, these findings suggest that PBC-FA wash water could serve as a safe and effective way to improve microbial safety of apples without affecting fruit quality.

## Data availability

Data will be made available on request.

## CRediT authorship contribution statement

**Victor Ryu:** Writing – original draft, Methodology, Investigation, Data curation, Conceptualization. **Piyanan Chuesiang:** Writing – review & editing, Data curation. **Joseph Uknalis:** Writing – review & editing, Methodology, Investigation, Data curation. **Helen Ngo:** Writing – review & editing, Funding acquisition. **Tony Jin:** Writing – review & editing, Methodology, Funding acquisition. **Xuetong Fan:** Writing – review & editing, Supervision, Project administration, Methodology, Funding acquisition, Conceptualization.

## Declaration of competing interest

Please check the following as appropriate.

o X Authors have participated in (a) conception and design, or (b) analysis and interpretation of the data; (c) drafting the article or revising it critically for important intellectual content; and (d) approval of the final version.
o X This manuscript has not been submitted to, nor is under review at, another journal or other publishing venue.
o X The authors have no affiliation with any organization with a direct or indirect financial interest in the subject matter discussed in the manuscript
o The following authors have affiliations with organizations with direct or indirect financial interest in the subject matter discussed in the manuscript:

## References

[1] Teng Z., Van Haute S., Zhou B., Hapeman C.J., Millner P.D., Wang Q., Luo Y. (2018). Impacts and interactions of organic compounds with chlorine sanitizer in recirculated and reused produce processing water. PLoS One.

[2] Estrada E.M., Hamilton A.M., Sullivan G.B., Wiedmann M., Critzer F.J., Strawn L.K. (2020). Prevalence, persistence, and diversity of *Listeria monocytogenes* and *Listeria* species in produce packinghouses in three US states. J. Food Protect..

[3] Fan X., Gurtler J.B., Mattheis J.P. (2023). Possible sources of *Listeria monocytogenes* contamination of fresh-cut apples and antimicrobial interventions during antibrowning treatments: a review. J. Food Protect. 86.

[4] Pietrysiak E., Ganjyal G.M. (2018). Apple peel morphology and attachment of *Listeria innocua* through aqueous environment as shown by scanning electron microscopy. Food Control.

[5] Burnett S.L., Beuchat L.R. (2002). Differentiation of viable and dead *Escherichia coli* O157: H7 cells on and in apple structures and tissues following chlorine treatment. J. Food Protect..

[6] Rodgers S.L., Cash J.N., Siddiq M., Ryser E.T. (2004). A comparison of different chemical sanitizers for inactivating *Escherichia coli* O157: H7 and *Listeria monocytogenes* in solution and on apples, lettuce, strawberries, and cantaloupe. J. Food Protect..

[7] McGlynn W. (2004). Guidelines for the use of chlorine bleach as a sanitizer in food processing operations. Oklahoma Cooperative Extension Service. https://extension.okstate.edu/fact-sheets/guidelines-for-the-use-of-chlorine-bleach-as-a-sanitizer-in-food-processing-operations.html.

[8] Gopal K., Tripathy S.S., Bersillon J.L., Dubey S.P. (2007). Chlorination byproducts, their toxicodynamics and removal from drinking water. J. Hazard Mater..

[9] Wang R.Y., Shen X., Su Y., Critzer F., Zhu M.-J. (2023). Chlorine and peroxyacetic acid inactivation of *Listeria monocytogenes* in simulated apple dump tank water. Food Control.

[10] Khan S., Beattie T.K., Knapp C.W. (2016). Relationship between antibiotic-and disinfectant-resistance profiles in bacteria harvested from tap water. Chemosphere.

[11] Tong C., Hu H., Chen G., Li Z., Li A., Zhang J. (2021). Chlorine disinfectants promote microbial resistance in *Pseudomonas* sp. Environ. Res..

[12] Liu S.-S., Qu H.-M., Yang D., Hu H., Liu W.-L., Qiu Z.-G., Shen Z.-Q. (2018). Chlorine disinfection increases both intracellular and extracellular antibiotic resistance genes in a full-scale wastewater treatment plant. Water Res..

[13] Zhang S., Wang Y., Lu J., Yu Z., Song H., Bond P.L., Guo J. (2021). Chlorine disinfection facilitates natural transformation through ROS-mediated oxidative stress. ISME J..

[14] Desbois A.P. (2012). Potential applications of antimicrobial fatty acids in medicine, agriculture and other industries. Recent Pat. Anti-Infect. Drug Discov..

[15] EFSA (2007). Opinion of the Scientific Panel on food additives, flavourings, processing aids and materials in contact with food (AFC) related to an application on the use of ethyl lauroyl arginate as a food additive. EFSA J..

[16] FDA (1997). Substances generally recognized as safe. Food and drug administration fed. Reg.

[17] Ryu V., Chuesiang P., Ngo H., Ashby R.D., Fan X. (2022). Sustainable bio-based antimicrobials derived from fatty acids: synthesis, safety, and efficacy. Crit. Rev. Food Sci. Nutr..

[18] Yoon B.K., Jackman J.A., Valle-González E.R., Cho N.-J. (2018). Antibacterial free fatty acids and monoglycerides: biological activities, experimental testing, and therapeutic applications. Int. J. Mol. Sci..

[19] Ngo H.L., Fox P.S., Nuñez A., Moreau R.A., Haas M.J. (2014). Catalytic synthesis and characterization of phenol‐branched‐chain fatty acid isomers. Eur. J. Lipid Sci. Technol..

[20] Fan X., Wagner K., Sokorai K.J., Ngo H. (2017). Inactivation of gram-positive bacteria by novel phenolic branched-chain fatty acids. J. Food Protect..

[21] Solans C., Morales D., Homs M. (2016). Spontaneous emulsification. Curr. Opin. Colloid Interface Sci..

[22] Busch S.V., Donnelly C.W. (1992). Development of a repair-enrichment broth for resuscitation of heat-injured *Listeria monocytogenes* and *Listeria innocua*. Appl. Environ. Microbiol..

[23] Espina L., García-Gonzalo D., Pagán R. (2016). Detection of thermal sublethal injury in *Escherichia coli* via the selective medium plating technique: mechanisms and improvements. Front. Microbiol..

[24] Yun J., Yan R., Fan X., Gurtler J., Phillips J. (2013). Fate of *E. coli* O157: H7, *Salmonella* spp. and potential surrogate bacteria on apricot fruit, following exposure to UV-C light. Int. J. Food Microbiol..

[25] Fan X., Mattheis J.P. (1998). BaggingFuji'Apples during fruit development affects color development and storage quality. Hortscience.

[26] Roy K., Popelier P.L. (2009). Predictive QSPR modeling of the acidic dissociation constant (pKa) of phenols in different solvents. J. Phys. Org. Chem..

[27] Arunmanee W., Pathania M., Solovyova A.S., Le Brun A.P., Ridley H., Baslé A., Lakey J.H. (2017). Gram-negative trimeric porins have specific LPS binding sites that are essential for porin biogenesis. Proceedings of the National Academy of Sciences.

[28] Choi U., Lee C.-R. (2019). Distinct roles of outer membrane porins in antibiotic resistance and membrane integrity in *Escherichia coli*. Front. Microbiol..

[29] Casillas-Vargas G., Ocasio-Malavé C., Medina S., Morales-Guzmán C., Del Valle R.G., Carballeira N.M., Sanabria-Ríos D.J. (2021). Antibacterial fatty acids: an update of possible mechanisms of action and implications in the development of the next-generation of antibacterial agents. Prog. Lipid Res..

[30] Wilhelm M.J., Gh M.S., Wu T., Li Y., Chang C.-M., Ma J., Dai H.-L. (2021). Determination of bacterial surface charge density via saturation of adsorbed ions. Biophys. J..

[31] Reay P.F. (1998). The effects of maturity on colour and concentration of pigments in the blush and shaded sides of ‘Gala’apple fruit during cool storage. J. Hortic. Sci. Biotechnol..

