# Supplementary material for: Bio-based phenolic branched-chain fatty acid in wash water reduced populations of Listeria innocua on apple fruit
Source: Heliyon. 2024 Jan 17;10(3):e24901. doi: 10.1016/j.heliyon.2024.e24901 (PMC10837536; doi:10.1016/j.heliyon.2024.e24901)
Supplement: Multimedia component 1 [file mmc1.docx]

# **Supplementary Table 1.** Difference in weight of the apple after treating for 5 min with different solution.

| Water (g) | 5% EtOH (g) | Chlorine (g) | PBC-FA (g) |
| --- | --- | --- | --- |
| 0.41 ± 0.007^A^ | 0.51 ± 0.008^B^ | 0.46 ± 0.042^AB^ | 0.52 ± 0.064^B^ |

***** Different capital letters (p ≤.05) were used to indicate significant differences between treatments

# **Supplementary Table 2.** L*, a*, b*, and ΔE values of shade and blush sides of apple fruit washed with different solutions. Color was measured on 1, 7 and 14 days of storage at 20°C after treatments.

| Shade side | Day | L* | a* | b* | ΔE |
| --- | --- | --- | --- | --- | --- |
| Control | 1 | 66.4 ± 2.7 ^ABa^ | 14.9 ± 2.9 ^Aa^ | 36.0 ± 1.5 ^Aa^ | 3.1 ± 1.8 ^Aa^ |
|  | 7 | 67.0 ± 1.5 ^Aa^ | 14.6 ± 1.9 ^Aa^ | 37.6 ± 1.8 ^Aab^ | 3.3 ± 2.3 ^Aa^ |
|  | 14 | 65.1 ± 2.5 ^ABa^ | 14.5 ± 2.1 ^CDa^ | 39.2 ± 1 ^ABb^ | 5.2 ± 3.1 ^Aa^ |
| Water | 1 | 66.1 ± 2.5 ^ABa^ | 14.0 ± 3.2 ^Aa^ | 33.8 ± 3.7 ^Aa^ | 5.5± 1.6 ^Aa^ |
|  | 7 | 65.3 ± 3.4 ^Aa^ | 16.3 ± 6.3 ^Aa^ | 36.0 ± 3.9 ^Aa^ | 7.1 ± 4.3 ^Aa^ |
|  | 14 | 68.0 ± 3.3 ^ABa^ | 13.6 ± 1.1 ^BCa^ | 40.1 ± 3.1 ^ABa^ | 5.7 ± 2.0 ^Aa^ |
| EtOH | 1 | 62.8 ± 3.5 ^Aa^ | 15.7 ± 2.9 ^Aa^ | 35.3 ± 3.8 ^Aa^ | 6.9 ± 5.1 ^Aa^ |
|  | 7 | 67.1 ± 4.3 ^Aa^ | 14.5 ± 4.4 ^Aa^ | 36.5 ± 2.2 ^Aa^ | 4.1 ± 3.3 ^Aa^ |
|  | 14 | 68.8 ± 1.7 ^ABa^ | 11.0 ± 1.9 ^Aa^ | 42.4 ± 1.9 ^Bb^ | 7.0 ± 1.3 ^Aa^ |
| Chlorine | 1 | 68.1 ± 2.6 ^Ba^ | 12.3 ± 2.6 ^Aa^ | 35.5 ± 1.4 ^Aa^ | 3.8 ± 0.6 ^Aa^ |
|  | 7 | 67.3 ± 1.4 ^Aa^ | 14.7 ± 1.7 ^Aab^ | 36.3 ± 2.3 ^Aa^ | 3.0 ± 1.0 ^Aa^ |
|  | 14 | 64.5 ± 3.4 ^Aa^ | 17.3 ± 2.0 ^Db^ | 38.0 ± 0.8 ^Aa^ | 5.9 ± 2.3 ^Aa^ |
| PBC-FA | 1 | 66.9 ± 1.3 ^ABa^ | 13.8 ± 3.7 ^Aa^ | 35.7 ± 1.2 ^Aa^ | 3.6 ± 1.9 ^Aa^ |
|  | 7 | 68.8 ± 2.6 ^Aa^ | 12.4 ± 4.3 ^Aa^ | 38.4 ± 4.4 ^Aab^ | 6.2 ± 0.9 ^Ab^ |
|  | 14 | 70.8 ± 3.4 ^Ba^ | 9.7 ± 1.8 ^ABa^ | 42.4 ± 2.1 ^Bb^ | 8.1 ± 0.6 ^Ab^ |

***** Different capital letters (p ≤.05) were used to indicate significant differences between treatments on corresponding days, while different lower-case letters were used to indicate significant differences (p ≤.05) between means on days within the treatment

| Blush side | Day | L* | a* | b* | ΔE |
| --- | --- | --- | --- | --- | --- |
| Control | 1 | 38.2 ± 3.7 ^Aa^ | 28.9 ± 2.6 ^Aa^ | 13.9 ± 2.3 ^Aa^ | 6.5 ± 3.6^Aa^ |
|  | 7 | 43.0 ± 3.3 ^Aa^ | 32.8 ± 1.8 ^Aa^ | 20.0 ± 2.2 ^Ab^ | 4.7 ± 1.1 ^Aa^ |
|  | 14 | 42.6 ± 1.9 ^Ba^ | 32.6 ± 1.1 ^Aa^ | 19.8 ± 0.6 ^ABb^ | 3.3 ± 0.6 ^Aa^ |
| Water | 1 | 42.1 ± 1.5 ^Aa^ | 32.7 ± 1.6 ^Ba^ | 18.8 ± 2.8 ^Ba^ | 3.0 ± 1.9 ^Aa^ |
|  | 7 | 45.6 ± 6.9 ^Aa^ | 29.6 ± 2.0 ^Ba^ | 20.8 ± 6.4 ^Aa^ | 8.9 ± 1.0 ^Aa^ |
|  | 14 | 43.9 ± 1.3 ^Ba^ | 31.2 ± 1.3 ^Aa^ | 22.3 ± 2.1 ^BCa^ | 5.5 ± 0.8 ^Aa^ |
| EtOH | 1 | 42.2 ± 1.8 ^Aa^ | 31.4 ± 1.9 ^ABa^ | 17.7 ± 1.0 ^Ba^ | 2.4 ± 4.9 ^ABa^ |
|  | 7 | 42.0 ± 2.0 ^Aa^ | 31.5 ± 1.0 ^ABa^ | 18.8 ± 3.5 ^Aa^ | 3.1 ± 2.1 ^Aa^ |
|  | 14 | 43.6 ± 0.8 ^Ba^ | 32.7 ± 0.8 ^Aa^ | 20.6 ± 1.5 ^ABa^ | 3.9 ± 2.2 ^Aa^ |
| Chlorine | 1 | 40.3 ± 2.1 ^Aa^ | 30.4 ± 1.4 ^ABa^ | 16.1 ± 1.1 ^ABa^ | 3.1 ± 3.4 ^ABa^ |
|  | 7 | 39.0 ± 1.4 ^Aa^ | 30.0 ± 0.8 ^ABa^ | 16.4 ± 0.8 ^Aa^ | 3.7 ± 1.0 ^Aa^ |
|  | 14 | 39.2 ± 2.5 ^Aa^ | 32.2 ± 2.0 ^Aa^ | 17.9 ± 3.2 ^Aa^ | 4.5 ± 2.3 ^ABa^ |
| PBC-FA | 1 | 42.3 ± 1.8 ^Aa^ | 33.0 ± 0.4 ^Ba^ | 18.5 ± 1.2 ^Ba^ | 2.1 ± 1.6 ^Ba^ |
|  | 7 | 43.8 ± 2.5 ^Aa^ | 32.4 ± 1.2 ^ABab^ | 21.1 ± 3.2 ^Aab^ | 4.7 ± 1.0 ^Aab^ |
|  | 14 | 45.8 ± 1.0 ^Ba^ | 30.8 ± 1.1 ^Ab^ | 24.7 ± 2.1 ^Cb^ | 8.4 ± 2.3 ^Bb^ |

***** Different capital letters (p ≤.05) were used to indicate significant differences between treatments on corresponding days, while different lower-case letters were used to indicate significant differences (p ≤.05) between means on days within the treatment

# **Supplementary Table 3.** Peak positive force and positive area of the apple treated with different solution.

|  | Day | Peak Positive Force (g) | Positive Area (g.sec) |
| --- | --- | --- | --- |
| Control | 0 | 5787 ± 692 ^a^ | 4557 ± 636 ^a^ |
|  | 1 | 6118 ± 851 ^Aa^ | 4948 ± 824 ^Aa^ |
|  | 7 | 5684 ± 292 ^Aa^ | 4457 ± 155 ^Aa^ |
|  | 14 | 5364 ± 582 ^Aa^ | 4082 ± 473 ^Aa^ |
| Water | 0 | 5787 ± 692 ^a^ | 4557 ± 636 ^a^ |
|  | 1 | 6134 ± 329 ^Aa^ | 4902 ± 208 ^Aa^ |
|  | 7 | 5361 ± 558 ^Aa^ | 4229 ± 369 ^Aa^ |
|  | 14 | 5663 ± 594 ^Aa^ | 4479 ± 507 ^Aa^ |
| EtOH | 0 | 5787 ± 692 ^a^ | 4557 ± 636 ^a^ |
|  | 1 | 5259 ± 358 ^Aa^ | 4289 ± 363 ^Aa^ |
|  | 7 | 5382 ± 287 ^Aa^ | 4196 ± 88 ^Aa^ |
|  | 14 | 5133 ± 108 ^Aa^ | 3950 ± 27 ^Aa^ |
| Chlorine | 0 | 5787 ± 692 ^a^ | 4557 ± 636 ^a^ |
|  | 1 | 5893 ± 566 ^Aa^ | 4780 ± 362 ^Aa^ |
|  | 7 | 5658 ± 328 ^Aa^ | 4535 ± 196 ^Aa^ |
|  | 14 | 5390 ± 379 ^Aa^ | 4123 ± 220 ^Aa^ |
| PBC-FA | 0 | 5787 ± 692 ^a^ | 4557 ± 636 ^a^ |
|  | 1 | 5752 ± 31 ^Aa^ | 4744 ± 98 ^Aa^ |
|  | 7 | 5332 ± 214 ^Aa^ | 4341 ± 264 ^Aa^ |
|  | 14 | 5656 ± 554 ^Aa^ | 4217 ± 203 ^Aa^ |

***** Different capital letters (p ≤.05) were used to indicate significant differences between treatments on corresponding days, while different lower-case letters were used to indicate significant differences (p ≤.05) between means on days within the treatment

| Day | Control | Water | 2.5% EtOH | Chlorine | PBC-FA |
| --- | --- | --- | --- | --- | --- |
| 0 | 14.1 ± 0.5 ^a^ | 14.1 ± 0.5 ^a^ | 14.1 ± 0.5 ^a^ | 14.1 ± 0.5 ^a^ | 14.1 ± 0.5 ^a^ |
| 1 | 14.3 ± 0.6 ^Aa^ | 14.2 ± 0.1 ^Aa^ | 13.8 ± 0.1 ^Aa^ | 14.4 ± 0.6 ^Aa^ | 14.1 ± 0.5 ^Aa^ |
| 7 | 14.2 ± 0.3 ^Aa^ | 14.0 ± 0.5 ^Aa^ | 14.3 ± 0.5 ^Aa^ | 14.3 ± 0.2 ^Aa^ | 14.2 ± 0.5 ^Aa^ |
| 14 | 14.1 ± 0.1 ^Aa^ | 14 ± 0 ^Aa^ | 14.1 ± 0.3 ^Aa^ | 14.1 ± 0.2 ^Aa^ | 14.3 ± 0.2 ^Aa^ |

# **Supplementary Table 4.** BRIX value of the apple juice extracted from apple that has gone through different treatments.

***** Different capital letters (p ≤.05) were used to indicate significant differences between treatments on corresponding days, while different lower-case letters were used to indicate significant differences (p ≤.05) between means on days within the treatment

# **Supplementary Figure 1**

**Supplementary Figure 1.** Changes in the average diameter of micelles in the stock PBC-FA emulsion during storage for 30 days at 4 and 20°C.

# **Supplementary Figure 2**

**Supplementary Figure 2.** Recovery of A) *L. innocua* and B) *E. coli* O157:H7 was observed for 24 hours in different concentrations of PBC-FA emulsions stored for 1 day (A1 and B1) and for 30 days at 4°C (A2 and B2) or 20°C (A3 and B3).
